# Molecular Diagnosis of Leishmaniasis in Spain: Development and Validation of Ready-To-Use Gel-Form Nested and Real-Time PCRs To Detect *Leishmania* spp.

**DOI:** 10.1128/spectrum.03354-22

**Published:** 2023-04-04

**Authors:** Carmen Chicharro, Javier Nieto, Silvia Miguelañez, Emilia Garcia, Sheila Ortega, Ana Peña, Jose Miguel Rubio, Maria Flores-Chavez

**Affiliations:** a Reference and Research Laboratory for Parasitology, National Centre for Microbiology, Instituto de Salud Carlos III (ISCIII), Madrid, Spain; b Centro de Investigación Biomédica en Red de Enfermedades Infecciosas (CIBERINFEC-ISCIII), Madrid, Spain; c WHO Collaborating Center for Leishmaniasis (WHOCCLeish), Madrid, Spain; d Fundación Mundo Sano, Madrid, Spain; Institut de Recherche pour le Développement

**Keywords:** leishmaniasis, molecular diagnosis, sensitivity, specificity, nested PCR, qPCR, parasite load, gel system

## Abstract

Leishmaniasis is an endemic parasitic disease in at least 98 countries. In Spain, it is considered a zoonosis caused by Leishmania infantum, with an annual incidence of 0.62 cases/100,000 inhabitants. The predominant clinical manifestations are the cutaneous (CL) and visceral forms (VL), and the diagnosis is performed by parasitological, serological, and molecular tests. At the WHO Collaborating Center for Leishmaniasis (WHOCCLeish), routine diagnostic tests are based on a nested PCR (Ln-PCR), culture, and serological tests. To simplify our PCR protocol, we aimed to develop and validate a ready-to-use nested gel-form PCR (LeishGelPCR) and a duplex real-time PCR (qPCR) that allowed simultaneous detection of *Leishmania* and mammalian DNA as an internal control (Leish-qPCR). Clinical validation was performed in 200 samples from the WHOCCLeish collection; 92 and 85 out of 94 and 87 samples were positive by LeishGelPCR and Leish-qPCR, respectively, showing a sensitivity of 98% in both approaches. The specificity was 100% for LeishGelPCR and 98% for Leish-qPCR. The limits of detection of both protocols were similar (0.5 and 0.2 parasites/reaction). Parasite loads in VL and CL forms were similar, although high loads were observed when invasive samples were tested. In conclusion, LeishGelPCR and Leish-qPCR showed excellent performance in the diagnosis of leishmaniasis. These new forms of 18S rRNA gene PCR are equivalent to Ln-PCR and can be introduced in the algorithm for CL and VL diagnosis.

**IMPORTANCE** Although the gold standard for diagnosis of leishmaniasis is the microscopic observation of amastigotes, molecular techniques are becoming a cost-efficient alternative. Currently, PCR is a routine resource that is used in many reference microbiology laboratories. In this article, we have described two ways to improve the reproducibility and usability of the molecular detection of *Leishmania* spp. These new approaches could be introduced even in middle- and low-resource laboratories; one is a ready-to-use gel-form system of a nested PCR and the other is a real-time PCR. We show why molecular diagnosis is the best methodology to confirm a clinical suspicion of leishmaniasis with higher sensitivity than traditional methods, thus facilitating early diagnosis and timely treatment of human leishmaniasis.

## INTRODUCTION

Leishmaniasis is a parasitic disease caused by protozoa of the genus *Leishmania* and is transmitted by sandflies (*Phlebotomus*) species. Leishmaniasis affects at least 98 countries and has four main forms of the disease: visceral leishmaniasis (VL; also known as kala-azar), post-kala-azar dermal leishmaniasis (PKDL), cutaneous leishmaniasis (CL), and mucocutaneous leishmaniasis (MCL) (1, 2). After the infective bite, the incubation period is quite variable, ranging from a few weeks to several months (3). CL, the most common form, can heal on its own, but it also can cause disfiguring skin lesions that can leave lifelong scars and/or evolve into MCL several months, or even years, after skin ulcers heal. The lesions can lead to the partial or total destruction of the mucosal membranes of the nose, mouth, and throat cavities and surrounding tissues (4). The most severe form of leishmaniasis is VL, causing fever, weight loss, and spleen and liver enlargement (2). It is often confused with other febrile illnesses, and its misdiagnosis may delay treatment and lead to the death of patients. Early diagnosis and complete treatment are crucial to avoid complications and reduce parasite transmission (5). Improvement of diagnostic tools for leishmaniasis diagnosis is part of the WHO response to control leishmaniasis in the world (2).

In Spain, leishmaniasis is a zoonosis caused by Leishmania infantum and is transmitted by female sandflies *Phlebotomus perniciosus* and *P. ariasi*, with dogs being the main reservoir (6). Human leishmaniasis is detected throughout the country throughout the year, with a reported average annual incidence of 0.62 cases per 100,000 inhabitants (7). In recent years, the spectacular outbreak of Fuenlabrada (Madrid), with more than 700 cases so far, has shown the impact of urbanization and the role of wild animals (hares) as reservoirs increasing the risk of transmission of L. infantum when they are surrounding urban areas (8–10). As L. infantum is the only species of *Leishmania* circulating in Spain, it is responsible for autochthonous leishmaniasis, including CL and VL, while PKDL is rare and is mostly described in immunocompromised patients due to HIV (11). In contrast, imported leishmaniasis is caused by different species depending on the geographical region where it was acquired, not observing a higher frequency of any specific species (12).

Diagnosis of leishmaniasis depends on the clinical manifestation and can be performed using bone marrow, whole blood, skin biopsy specimens, or any other sample. Microscopic observation (MO) of *Leishmania* amastigotes is considered the gold standard; the execution time is short (approximately 1 h [considering the staining and observation time]), but its sensitivity and specificity are limited because it depends on the parasite load and the operator’s skills (3, 5, 13). Additionally, MO does not allow distinction between *Leishmania* species since morphological differences, if any, are negligible. Culture increases parasite detection, but it is labor-intensive. In certain circumstances, tests based on anti-*Leishmania* antibodies can help to confirm suspected cases, but, in general, molecular techniques have higher sensitivity (Table 1). For that reason, DNA detection by PCR became the alternative reference test for leishmaniasis diagnosis, even minimizing the use of invasive samples such as blood instead of bone marrow in the case of VL diagnosis (14, 15). Our lab, as a WHO Collaborating Centre for Leishmaniasis, offers support to confirm clinical suspicion using parasitological, serological, and molecular tests (6). According to different studies performed in our lab, nested PCR, targeting the small subunit 18S rRNA gene (Ln-PCR), was the technique for *Leishmania* diagnosis that presented the greatest sensitivity and specificity, but it is tedious and needs two amplifications with the increased chance of contamination (15). Therefore, our work aimed to develop and validate a ready-to-use gel-form system of Ln-PCR (LeishGelPCR) and a real-time PCR (qPCR; Leish-qPCR) based on the same target to reduce the disadvantages of the original method.

**TABLE 1:** Characteristics of the main parasitological, serological, and molecular diagnostic techniques for the diagnosis of human leishmaniasis in Spain by place of use

Test	Run time	Sample	Sensitivity	References	Place^*a*^
Microscopic observation (MO)	1 h	Bone marrow	67 to 73%	15, 33, 34	Primary health care centers (PHCC)
		Biopsy specimens	36 to 65%	30, 35	
Culture	1 mo	Bone marrow	36 to 66%	33, 34, 36	Secondary health care centers (SHCC); Tertiary health care centers (THCC)
		Whole blood	61 to 92%	36	
Polymerase chain reaction (PCR)	6 h	Bone marrow	100%	14, 15	SHCC; THCC
		Whole blood	79 to 100%	14, 15, 36	
		Biopsy specimens	92.5%	35	
Immunofluorescence indirect antibody test (IFAT)	4 h	Serum/plasma	63 to 84%	29, 33	SHCC; THCC
rk39 immunochromatographic test (rk39 ICT)	20 min	Serum/plasma	67 to 83%	29	PHCC; SHCC; THCC

a PHCC, primary health care centres; SHCC, secondary health care centres; THCC, tertiary health care centres.

## RESULTS

### Development of LeishGelPCR and Leish-qPCR.

LeishGelPCR, the ready-to-use gel form, was developed using the same protocols and reagents as Ln-PCR. The analytical sensitivity experiments testing spiked samples, which were adjusted at 5 × 10^6^ to 0.05 parasite equivalents/reaction, revealed that the first reaction of LeishGelPCR was more sensitive than the first reaction of Ln-PCR (0.5 parasites versus 5,000 parasites). However, this improvement was not noticed in the second reaction; both LeishGelPCR and Ln-PCR yielded a positive result at 0.5 parasite equivalents per reaction (Fig. S1 in the supplemental material). Ten repetitive tests of 5, 0.5, and 0.05 parasite equivalents/reaction showed that the limits of detection (LOD) of LeishGelPCR and Ln-PCR (comparing the final results, that is, results of the second reactions) were 0.5 and 5 parasite equivalents per reaction, respectively (Table S1).

Through similar experiments, using spiked samples adjusted to 1 × 10^6^ to 0.001 parasite equivalents/reaction (Fig. S2) and eight repetitive tests of 1, 0.1, 0.01, and 0.001 parasite equivalents/reaction, the LOD of Leish-qPCR using PROBIT analysis was 0.2 parasites/reaction (95% confidence interval [CI 95%]: 0.1 to 1.1 parasite equivalents/reaction) (Table S2).

### Clinical validation of LeishGelPCR and Leish-qPCR.

To validate the new modifications of *Leishmania* PCR, 10 DNA samples from leishmaniasis patients with positive results by Ln-PCR, 5 at least since the first reaction, were included; all samples returned positive results by LeishGelPCR and Leish-qPCR (Table S3). To explore specificity and cross-amplification, DNA samples from uninfected individuals with negative results by Ln-PCR or positive results for other parasitic infections and culture of Mycobacterium tuberculosis were tested, and all samples yielded negative results in all *Leishmania* PCRs (Table S4).

**FIG 1:**
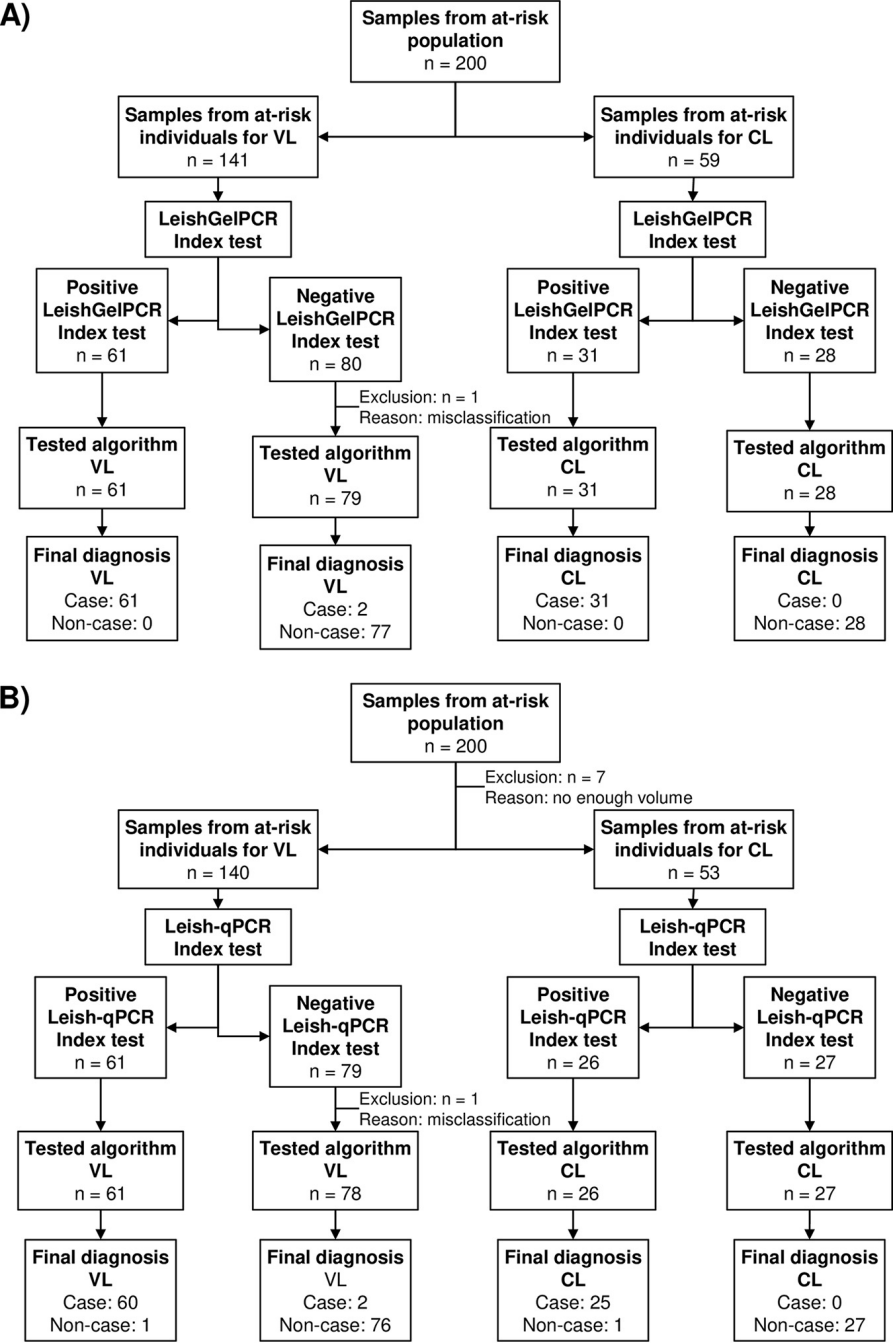
(A and B) Diagram of samples and testing workflow used to evaluate the performance of LeishGelPCR (A) and Leish-qPCR (B).

Once the newly established conditions were shown to work, we launched a new validation study including a larger number of samples (*n* = 200). The demographic baseline data and positivity/negativity by different tests for leishmaniasis diagnosis are described in Table 2. A total of 200 samples from an at-risk population to be infected with *Leishmania* were tested by LeishGelPCR, 141 samples with a suspected VL and 59 with a suspected CL (Fig. 1A). Sixty-one out of 63 positive samples for VL and all 31 for CL were identified with LeishGelPCR. Repetition by Ln-PCR of the two VL discrepant samples (D164 and D13) confirmed the negative results of LeishGelPCR (Table 3). All samples from uninfected individuals were successfully identified as negative. One case (sample E^1^35) of this last group was excluded from the final analysis due to the initial misclassification, although all PCRs returned negative results; the rk39 immunochromatographic test (ICT) and the indirect immunofluorescence antibody test (IFAT) showed positive results (Table 3). This patient was in follow-up due to VL 14 years ago and had an immunosuppressed condition.

**TABLE 2:** Characteristics of samples at baseline

Epidemiological, sample type and laboratory results information	VL suspicion		CL suspicion	
	Infected *n* = 63	Uninfected *n* = 78	Infected *n* = 31	Uninfected *n* = 28
Age				
Median (IQR)^*a*^	43 (38 to 56)	55 (37 to 69)	49 (9 to 61)	60 (39 to 72)
Origin, *n* (%)				
Spain	44 (70)	49 (63)	13 (42)	22 (79)
Others	7 (11)	5 (6)	5 (16)	0 (0)
Unknown	12 (19)	24 (31)	13 (42)	6 (21)
Gender, *n* (%)				
Female	15 (24)	26 (33)	15 (48)	11 (39)
Male	48 (76)	52 (67)	16 (52)	17 (61)
Sample, *n* (%)				
Whole blood	37 (59)	44 (56)		
Bone marrow	24 (38)	33 (42)		
Others	2 (3)	1 (1)		
Fresh tissue biopsy specimens			27 (87)	25 (89)
FFPE tissue specimens			4 (13)	3 (11)
Culture, *n* (%)				
Positive	17 (31)		8 (40)	
Negative	38 (69)	75 (100)	12 (60)	25 (100)
Ln-PCR, *n* (%)				
Positive	63 (100)		31 (100)	
Negative		78 (100)		28 (100)

a IQR, interquartile range.

**TABLE 3:** Characteristics of samples with discrepant results and those that were excluded from comparisons

Sample^*a*^		Results at baseline^*b*^^,^^*c*^				Results of this study^*b*^^,^^*c*^					
Code	Matrix	rK39 ICT	IFAT	Culture	Ln-PCR^*d*^	Ln-PCR^*e*^	LeishGelPCR		Leish-qPCR^*f*^		IC
							First	Second	First	Second	
D164	WB	**P**	ND	N	**P**	N	N	N	**37.74**	**37.53**	17.49
D 60	WB	**P**	**1/160**	N	**P**	N	**P**	N	N	N	16.23
D 13	BM	NA	NA	N	**P**	N	N	N	N	N	15.53
D112	FTB	NA	NA	N	N	**P**	N	N	**39.07**	**38.51**	18.06
E^1^35	WB	**P**	**1/80**	N	N	N	N	NA	N	NA	15.22
E^2^ 64	BM	**P**	ND	**P**	**P**	NA	**P**	NA	ND	NA	NA
E^2^ 16	FTB	NA	NA	N	**P**	NA	**P**	NA	ND	NA	NA
E^2^ 18	FTB	NA	NA	N	**P**	NA	**P**	NA	ND	NA	NA
E^2^ 34	FTB	NA	NA	N	**P**	NA	**P**	NA	ND	NA	NA
E^2^156	FTB	NA	NA	ND	**P**	NA	**P**	NA	ND	NA	NA
E^2^197	FTB	NA	NA	ND	**P**	NA	**P**	NA	ND	NA	NA
E^2^198	FTB	NA	NA	ND	**P**	NA	**P**	NA	ND	NA	NA

a D, discrepant; E^1^, excluded sample in performance evaluation of both LeishGelPCR and Leis-qPCR, this sample should not be classified as uninfected; E^2^, excluded samples in performance evaluation of Leish-qPCR, volume was not enough to run qPCR; WB, whole blood; FTB, fresh tissue biopsy specimen; BM, bone marrow.

b ICT, immunochromatographic test; IFAT, indirect immunofluorescence antibody test; P, positive; N, negative; NA, not applicable; ND, not done.

c Positive results in each test are highlighted in bold.

d Ln-PCR, previous result.

e Ln-PCR, new result.

f Leish-qPCR, positive results are expressed as threshold cycle (*C_T_*); IC, internal control.

A total of 193 samples were tested by Leish-qPCR. Due to low sample volume, seven specimens from at-risk populations were excluded from evaluation. Thus, 140 were from a suspected VL, and 53 were from a suspected CL (Fig. 1B). Sixty out of 62 positive samples for VL and all 25 for CL were successfully identified by Leish-qPCR. The positive samples yielded threshold cycle (*C_T_*) values ranging from 17.5 to 42.3 for the *Leishmania* probe. From 106 negative specimens, 104 were identified as negative. For the final analysis, the same excluded negative case (sample E^1^35) for LeishGelPCR was not included (Table 3). Repetition by Ln-PCR of the four discrepant samples (D164, D60, D13, and D112) showed negative results in samples with previously positive results and positive results in the previously negative sample (Table 3). These observations were attributed to *Leishmania* DNA content that was close to the LOD and adverse events due to freezing and thawing.

We want to highlight that 11 samples from patients with imported leishmaniasis who were infected with L. major (*n* = 9) and *L. braziliensis* (*n* = 2) tested positive by LeishGelPCR similar to Ln-PCR. These samples were also positive by Leish-qPCR, with the exception of two; they were not analyzed due to low sample volume (Data Set S1).

### Sensitivity and specificity of LeishGelPCR.

The global sensitivity, specificity, and accuracy of LeishGelPCR were 98, 100, and 99%, respectively (Table 4). The differences concerning Ln-PCR were not statistically significant (McNemar’s test = 0.5 and *P* = 0.5).

**TABLE 4:** Performance of LeishGelPCR and Leish-qPCR estimated by binomial analysis

Index test	Infection status		Sensitivity^*a*^			Specificity^*a*^			Accuracy^*a*^		
	Positive	Negative	%	95% CI		%	95% CI		%	95% CI	
Overall cases											
LeishGelPCR											
Positive	92	0	97.9	95.2	99.7	100	96.6	100	99.0	96.4	99.9
Negative	2	105									
Leish-qPCR											
Positive	85	2	97.7	91.9	99.7	98.1	93.3	99.8	97.9	94.8	99.4
Negative	2	103									
Cutaneous leishmaniasis											
LeishGelPCR											
Positive	31	0	100	88.8	100	100	87.8	100	100	93.9	100
Negative	0	28									
Leish-qPCR											
Positive	25	1	100	86.3	100	96.4	81.7	99.9	98.1	89.9	100
Negative	0	27									
Visceral leishmaniasis											
LeishGelPCR											
Positive	61	0	96.8	89.0	99.6	100	95.3	100	98.6	95.0	99.8
Negative	2	77									
Leish-qPCR											
Positive	60	1	96.8	88.8	99.6	98.7	93.0	100	97.8	93.8	99.6
Negative	2	76									

a According to McNemar’s test, the differences were not statistically significant (*P* > 0.05).

### Sensitivity and specificity of Leish-qPCR.

The global sensitivity, specificity, and accuracy were around 98%, and the differences concerning Ln-PCR were not statistically significant using McNemar’s test (0.0001; *P* = 1) (Table 4).

### Receiver operating characteristic (ROC) curve of Leish-qPCR.

**FIG 2:**
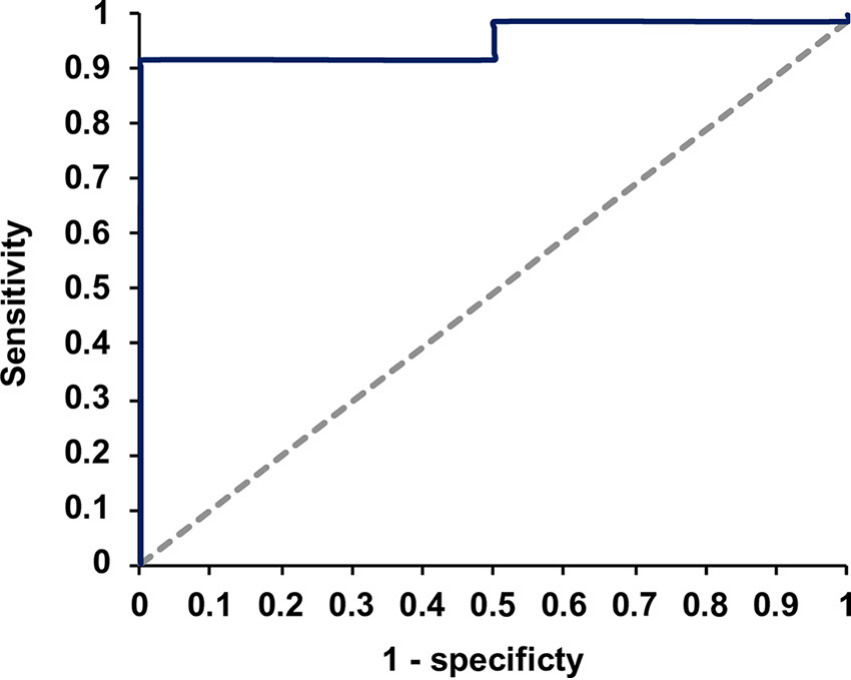
ROC curve of Leish-qPCR showing an AUC value of 0.953 (CI 95%: 0.890 to 1; *P* < 0.001).

According to the ROC curve analysis that included data from patients with *C_T_* values (*n* = 87), the area under the curve (AUC) value was 0.953 (CI 95%: 0.890 to 1; *P* < 0.001); that is, the performance of Leish-qPCR was outstanding. Then, with a *C_T_* value of 37.03 as a threshold, the sensitivity of Leish-qPCR was 92%, and the specificity was 100% (Fig. 2). Whereas, with a *C_T_* value of 43.28, the sensitivity and specificity were 100% and 97.7%, respectively.

### Parasite load estimations.

**FIG 3:**
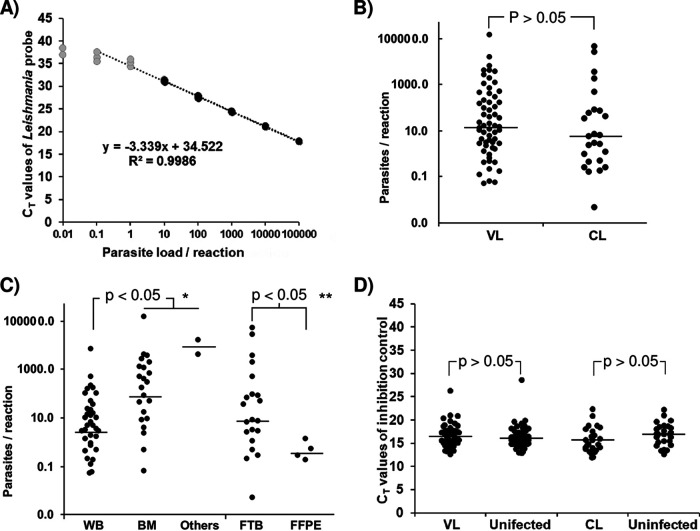
Leish-qPCR. (A) Standard curve built by linear regression; black dots, *C_T_* values included in the calculation of the equation to estimate parasite load; gray dots, *C_T_* values with the coefficient of variation higher than 25%. (B) Estimations of parasite load considering clinical form; VL, visceral leishmaniasis; CL, cutaneous leishmaniasis. According to nonparametric tests, the differences between medians were not statistically significant (Kruskal Wallis test = 1.167; *P* = 0.280). (C) Parasite load according to DNA matrix; WB, whole blood; BM, bone marrow; FTB, fresh tissue biopsy specimens; FFPE, formalin-fixed paraffin-embedded tissue specimens; *, Kruskal-Wallis test = 15.097, *P* < 0.001; **, Mann-Whitney *U* test = 16, *P* = 0.048. (D) *C_T_* values of the internal control according to clinical condition. Differences were not significant; Mann-Whitney *U* test = 4800.5, *P* = 0.624. Horizontal lines correspond to median values.

We built the standard curve by linear regression; the linearity was maintained up to 10 parasite equivalents/reaction (Fig. 3A). Using the equation *y* = −3.339*x* + 34.522 with an *R*^2^ of 0.9986 (*P* < 0.001), we extrapolated *C_T_* values in parasite equivalents/reaction, so the parasite load fluctuated from 0.1 to 1.3 × 10^5^ parasite equivalents/reaction. In 49% of samples (43/77), the parasite load was above 10 parasite equivalents/reaction. The median values (interquartile ranges) in VL and CL were 13.9 (2.4 to 215) and 5.9 (0.5 to 73) parasite equivalents/reaction with maximum values of 1.3 × 10^5^ and 4.4 × 10^4^ parasite equivalents/reaction, respectively (Fig. 3B). According to the nonparametric test, the differences between medians were not statistically significant (Kruskal Wallis test = 1.167; *P* = 0.280). However, considering the sample type, the parasite load median in VL was higher in bone marrow and lymphatic node aspirate/throat biopsy specimens (213.17 and 8,958.72 parasite equivalents/reaction, respectively) than in whole blood (4.92 parasite equivalents/reaction; Kruskal Wallis test = 15.097; *P* < 0.001). Similarly, the median parasite load in CL was higher in fresh tissue biopsy specimens (6.91 parasite equivalents/reaction) than in formalin-fixed paraffin-embedded (FFPE) tissue specimens (0.36 parasite equivalents/reaction; Mann-Whitney *U* test = 16; *P* = 0.048) (Fig. 3C).

Amplification of the human 18S rRNA gene showed no failures in Leish-qPCRs. The median *C_T_* value of internal controls (ICs) in 33 repetitions of a fresh blood sample from one volunteer blood donor was 18.4 ± 0.2, similar to the retrospective blood samples, which yielded a median *C_T_* value of 16.73 ± 1.6 (*n* = 81). The median *C_T_* value in the bone marrow was 16.01 ± 2.7 (*n* = 56), the median *C_T_* value in fresh tissue biopsy specimens was 15.73 ± 2.4 (*n* = 46), the median *C_T_* value in FFPE tissue specimens was 19.01 ± 2.5 (*n* = 7), and the median *C_T_* value in other types of matrices was 13.7 ± 1 (*n* = 3). These differences were not significant between infected and uninfected (Mann-Whitney *U* test = 4,800.5; *P* = 0.624) (Fig. 3D), except in fresh tissue biopsy specimens (Mann-Whitney *U* test = 356.5; *P* = 0.038).

### Correlation between diagnostic and index tests.

To evaluate the correlation patterns between the diagnostic and the index tests, we removed the results of the samples that could not be analyzed by Leish-qPCR and those that yielded discrepancies. Then, 84 samples were positive by all three molecular approaches; out of these, four were paraffin-embedded samples, so they were only studied by Ln-PCR and the new tests. Out of the remaining 80 samples, 75 were analyzed using another technique, either a serological test or a parasitological test depending on the type of sample (Table 5). In VL patients diagnosed by testing whole blood, anti-*Leishmania* IgG was detected in 93% (26/28) of cases by IFAT, whereas rk39 ICT returned positive in 77% (27/35) of cases only. Considering overall samples, culture returned a positive result in 35% (24/68) of specimens. The positivity rates of culture in blood and bone marrow samples were similar (31% [9/29] and 30% [6/20], respectively), whereas in fresh tissue biopsy specimens, this rate was slightly higher 47% (8/17). In the other specimens (lymph node aspirate and pharynges biopsy specimen), one returned positive and one returned negative (Table 5). However, these differences were not significant (χ^2^ = 1.696; *P* = 0.638).

**TABLE 5:** The observed frequency of the agreement profiles and their relationship with parasite load from positive cases by serological, parasitological, and molecular tests

Type of sample^*a*^	IFAT^*b*^^,^^*c*^	rk39^*b*^^,^^*c*^	Culture^*b*^^,^^*c*^	LnPCR^*b*^^,^^*c*^	LeishGelPCR^*b*^^,^^*c*^	Leish-qPCR^*b*^^,^^*c*^	*n* ^*d*^	Parasites/reaction		Positivity rate of culture, *n*/total (%)
								Minimum	Maximum	
WB	−	−	−	**+**	**+**	**+**	1	0.2		
	40	−	−	**+**	**+**	**+**	1	2.8		
	**80**	−	**+**	**+**	**+**	**+**	1	6.10^3^		
	**80**	**+**	**+**	**+**	**+**	**+**	1	11.3		
	**160**	−	−	**+**	**+**	**+**	1	0.6		
	**160**	**+**	−	**+**	**+**	**+**	6	0.1	447.8	
	**>160**	−	ND	**+**	**+**	**+**	1	45.7		
	**>160**	−	**+**	**+**	**+**	**+**	1	4.1		
	**>160**	**+**	ND/−	**+**	**+**	**+**	11	0.4	31.3	
	**>160**	**+**	**+**	**+**	**+**	**+**	4	4.1	136.8	
	ND	−	−	**+**	**+**	**+**	2	<0.1^*e*^	1.2	
	ND	**+**	ND/−	**+**	**+**	**+**	3	0.2	89.2	
	ND	**+**	**+**	**+**	**+**	**+**	2	1.5	5.7	9/35 (25.7)
BM	NA	NA	ND/−	**+**	**+**	**+**	16	0.1	1.10^5^	
	NA	NA	**+**	**+**	**+**	**+**	6	8.0	4.10^3^	6/22 (27.3)
Other	NA	NA	−	**+**	**+**	**+**	1	1.10^4^		
	NA	NA	**+**	**+**	**+**	**+**	1	4.10^3^		1/2 (50.0)
FTB	NA	NA	ND/C/−	**+**	**+**	**+**	13	<0.1^*e*^	4.10^4^	
	NA	NA	**+**	**+**	**+**	**+**	8	2.5	3.10^3^	8/21 (38.1)
FFPE	NA	NA	NA	**+**	**+**	**+**	4	0.2	1.2	
Positivity rate, *n*/total (%)	26/28 (92.8)	27/35 (77.1)	24/68 (35.3)	84/84 (100)						

a WB, whole blood; BM, bone marrow; FTB, fresh tissue biopsy specimen; FFPE, formalin-fixed paraffin-embedded sample.

b +, positive result; −, negative result; ND, not done; NA, not applicable; C, contaminated.

c Positive results in each test are highlighted in bold and gray background.

d *n*, number of patients.

e < 0.1, not-quantifiable value.

## DISCUSSION

For early and proper treatment against *Leishmania*, parasite detection is essential to confirm a clinical suspicion. When parasite load is high, amastigotes can be revealed by microscopy in a few minutes, but, although this technique is the gold standard, the performance of microscopic observation depends on the operator’s expertise (13). For 20 years, Ln-PCR was the standard molecular test of the WHO Collaborating Centre for Leishmaniasis in Spain; but to simplify this PCR protocol, we have developed a ready-to-use gel-form system and a real-time PCR using the same target of the nested conventional PCR (Ln-PCR) (15, 16). The performance of these new systems was equivalent to Ln-PCR, the accuracy of both approaches was high (between 98% and 100%), and they can be used interchangeably (Table 4). As the manipulation and operating time was shorter than Ln-PCR, the response time was improved; we gained approximately 30 min with the gel-form system and 3 h with qPCR. Furthermore, because Leish-qPCR includes the primers and probe to determine the mammalian 18S rRNA gene as a housekeeping target in the same reaction tube, we could assess DNA quality and PCR success at the same time (Fig. S2 in the supplemental material).

In the literature, different molecular tests to detect *Leishmania* DNA were described. These tests are mainly based on kinetoplast DNA (kDNA), 18S rRNA, internal transcribed spacer (ITS), and heat shock protein 70 (HSP70) (17, 18). According to a multicenter study conducted between different reference labs from Europe, the United States, and Asia, the qPCR targeting the minicircle of kDNA was the molecular approach with the highest sensitivity to detect all species of *Leishmania* parasites (16). Similar results were obtained in a Colombia study, but the kDNA primers presented cross-amplification with DNA from Trypanosoma cruzi and M. tuberculosis, showing its limitation in terms of specificity (17). The same study revealed that 18S rRNA qPCR presented the best performance in terms of analytical sensitivity and specificity. However, authors from Brazil described a qPCR based on the 18S rRNA gene that showed cross-amplification with other trypanosomatids, including T. cruzi and *T. rangeli*, among others (19). The primers in the Brazilian study were different from the Colombian ones and ours. We used primers described by Van Eys (20) because these primers flank an exclusive region of the 18S rRNA gene from the *Leishmania* genus. According to our evaluation of analytical specificity, primers R223 and R332 showed no cross-amplification with T. cruzi nor with M. tuberculosis.

In terms of analytical sensitivity, considering that the number of minicircles per parasite is 10^4^ copies (21), it is plausible that qPCRs based on kDNA yielded higher sensitivity than other targets with a lower number of copies per parasite. In fact, the 18S rRNA gene has about 166 copies in the *Leishmania* genome (22); therefore, it is necessary to use a nested PCR to achieve the same sensitivity as kDNA (14). The main disadvantages of a nested PCR are the number of steps that should be performed to run it and the multiple manipulations involving a high risk of cross-contamination; therefore, the use of nested PCR is not widely extended (13). A ready-to-use gel-form LeishGelPCR is an alternative system to reduce those inconveniences while keeping a low cost per reaction (double compared to Ln-PCR).

The first approaches of qPCRs included the addition of SYBR green as a reporter, like in the Colombia study (17). However, *Leishmania* identification and quantification with SYBR green using bone marrow samples can be complicated due to the high quantity of total genomic DNA from this kind of sample. In some cases, following dilution, identification and quantification can be achieved, but in samples with a low parasite load, *Leishmania* detection fails. Therefore, in this study, this drawback was resolved by designing a specific probe to detect *Leishmania* DNA into the fragment flanked for the same primers of nested PCR; thus, we improved sensitivity and specificity and achieved a similar LOD as Ln-PCR. According to our *in silico* analysis and experiments, no cross-amplification with T. cruzi was found, as Van Eys et al. previously reported. This fact is important because Chagas disease is one of the main imported parasitic infections in Spain (23). Due to the coronavirus disease 2019 (COVID-19) pandemic, real-time PCR equipment was implemented in most reference centers, even in county hospitals, and although the qPCR price is four times as much as Ln-PCR (without considering the DNA extraction cost), this increase was worth it since the manipulation and the probability of contamination were lower and the running time was shorter.

With a proper method of DNA extraction, different matrices of samples can be analyzed by these new forms of PCRs (Fig. 3C and D; Table 5), meaning that the 18S rRNA gene allows *Leishmania* detection similar to kDNA qPCR (24, 25). The success of detecting *Leishmania* DNA in each sample depends on the parasite load and the distribution of the parasites in the lesion in CL (26). Nonetheless, at least DNA equivalent to one amastigote must be present in the sample aliquot to be processed for DNA purification. Considering the characteristics of the infection outcome, more than one amastigote is likely to be found if the sample was properly taken. Therefore, the LOD of both LeishGelPCR and Leish-qPCR (0.5 and 0.2 parasites/reaction, respectively) is enough to determine *Leishmania* infection, even when it is caused by species other than L. infantum. In imported leishmaniasis caused by L. major and *L. braziliensis*, the newly developed molecular tests returned positive results like Ln-PCR (Data Set S1), since primers and probe detect the *Leishmania* genus (Fig. S3).

Currently, measurement of parasite load is not used to determine the therapeutic scheme of either VL or CL patients. The parasite load may vary over time and reflects both the interactions of the host with the parasite and the initial load that was inoculated during the fly bite (13). The usefulness of this information is controversial. In our series of samples, high parasite load could explain the success of isolating *Leishmania* by culture (Table 5); others found that parasite load in blood correlated positively with parasitic splenic score (27). Both examples maintain that molecular detection of *Leishmania* in the blood is useful because it is more sensitive than culture and avoids invasive sampling. However, both low and high parasite loads are related to active leishmaniasis (Fig. 3B). Similar results were described in Italy using kDNA qPCR (28). The comparisons are difficult because there is no consensus on standard curve building and the units of the extrapolation. We keep parasites per reaction as the unit of parasite load because only in blood samples could this unit be inferred to parasites per milliliter.

Despite the greater sensitivity of Ln-PCR, LeishGelPCR, and Leish-qPCR than other diagnostic tests (Table 5), parasitological and serological techniques are useful in other circumstances. For example, when parasite load is not uniform and discrepancies are observed (Table 3), serology confirms a clinical suspicion of a primoinfection (Table 5). Previously, we described that rk39 ICT showed lower sensitivity than IFAT (29). However, this technique returns a result in 10 min, while IFAT needs 3 h, Ln-PCR needs 8 h, LeishGelPCR needs 7 h, and Leish-qPCR needs 4 h. If the local laboratory does not have these capacities, the time of sample shipment must be added. In our experience, 100% of leishmaniasis cases are detected by a combination of molecular techniques with parasitological and/or serological tests. Finally, for interpretation of the result set, especially when there are discrepancies, epidemiological information is crucial to defining the case accurately.

**FIG 4:**
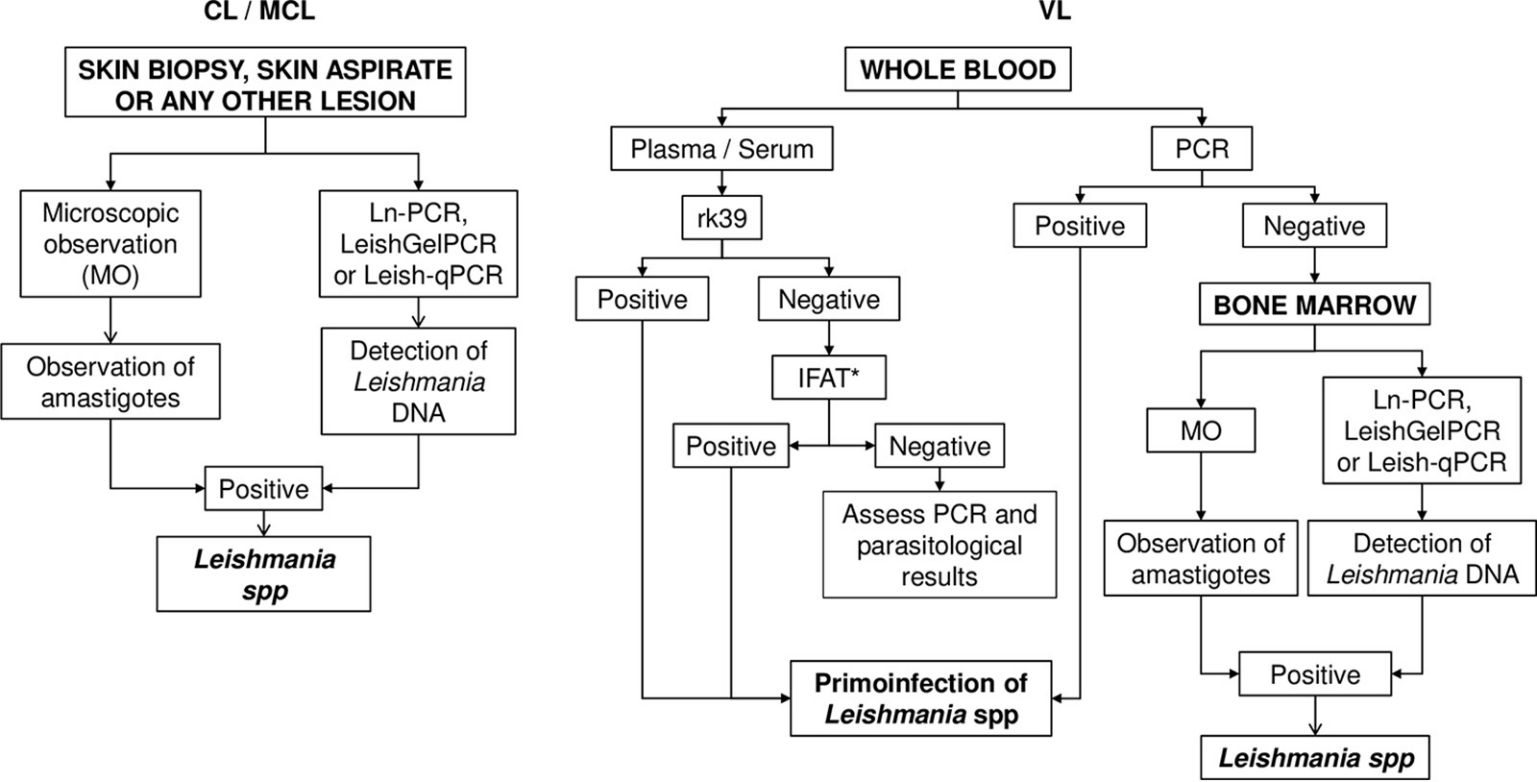
Proposal of an algorithm for the diagnosis of different clinical forms of leishmaniasis; *, IFAT could be replaced with another serological test to determine IgG anti-*Leishmania*.

The main limitation of our study is the retrospective design. Nonetheless, with convenient sampling, we tried to mimic the real situation of leishmaniasis in Madrid, Spain (10). In our routine, discrepant results are also observed. In this study, the negative results in positive samples could be attributed to poor DNA quality after freezing and thawing (Table 3). In fresh samples, discrepant results in duplicates are related to very low parasite load, close to the LOD of molecular tests. To resolve this drawback, new DNA purification from a new aliquot sample is useful. Another limitation is that the samples are exclusively from individuals living in Spain. Although the species of *Leishmania* is unknown (Data Set S1) in most of the VL and CL patients, L. infantum is the main etiological pathogen. Few samples with imported species of *Leishmania* were included, and they tested positive as in Ln-PCR. Besides this, a Colombian study showed that the *Leishmania* primers are useful and have good performance in detecting *Leishmania* species that are circulating in the United States (17). Nonetheless, prospective studies in different laboratories are needed to validate this research. Despite this, considering previous and present work, the algorithm for leishmaniasis diagnosis in a similar epidemiological scenario could be as shown in Fig. 4.

In addition, awareness in health professionals and the population is important so that CL can be recognized and diagnosis and treatment can be improved (30). Similarly, despite VL being considered the most severe form of *Leishmania* infection, in Spain, with an early diagnosis and timely treatment, the mortality rate is low, and a cure is achieved in all immunocompetent patients or disease is conveniently controlled in patients with immunosuppression (10).

In conclusion, LeishGelPCR and Leish-qPCR showed excellent performance in the diagnosis of leishmaniasis. These new forms of 18S rRNA gene PCR are equivalent to Ln-PCR and can be introduced in the algorithm of CL and VL diagnosis in similar epidemiological scenarios as in Spain. The gel-form allows for homogeneity of the PCRs, avoiding differences between days, operators, and laboratories. Both the gel-form PCR and qPCR reduce reagent handling, and, thus, the likelihood of contamination is also lower. In addition, Leish-qPCR returns results about the parasite load, but a harmonization of how to build a standard curve would be necessary for comparisons between different studies and assessment of whether its determination is worthwhile.

## MATERIALS AND METHODS

### Study design.

The development of LeishGelPCR and Leish-qPCR (experimental study) was performed using spiked DNA from whole-blood samples with *Leishmania* DNA from cultures. Exploratory validation of the new conditions was evaluated with 10 DNA samples from leishmaniasis patients (Table S3 in the supplemental material), 21 DNA samples from uninfected or infected individuals with other parasites (Table S4), and two DNA samples from Mycobacterium tuberculosis cultures.

For main clinical validation of the new techniques (i.e., to determine their sensitivity and specificity), a retrospective study following a case-control model was designed. Series were formed by convenience sampling from the clinical specimen collections of the Centre for Microbiology (CNM), Instituto de Salud Carlos III (ISCIII). The time frame of sample and data collection was between 2016 and 2017 (Table 6).

**TABLE 6:** Characteristics of sample series included to determine sensitivity and specificity of new PCR for *Leishmania* detection

Group	Matrix^*a*^	No. of samples	Clinical information	Previous results^*a*^	Yr of collection	Condition of storage
Cutaneous leishmaniasis (CL)	Fresh tissue biopsy specimens	27	Skin lesion	Ln-PCR + Culture +	2016	Frozen
	FFPE tissue specimens	4		Ln-PCR +		
Visceral leishmaniasis (VL)	Whole blood	37	Fever, hepatosplenomegaly	Ln-PCR + Culture + rK39 + /IFAT +	2016	Frozen
	Bone marrow	24		Ln-PCR + Culture +		
	Lymph node aspirate and pharynges biopsy specimen	2		Ln-PCR + Culture +		
Uninfected	Fresh tissue biopsy specimens	25	Skin lesion	Ln-PCR – Culture –	2017	Refrigerated or frozen
	FFPE tissue specimens	3		Ln-PCR –		
	Whole blood	44	Fever, hepatosplenomegaly	Ln-PCR – Culture – rK39 – /IFAT –		
	Bone marrow	33		Ln-PCR – Culture –		
	Lymph node aspirate	1		Ln-PCR – Culture –		

a FFPE, formalin-fixed paraffin-embedded tissue specimens; +, positive; –, negative.

### Clinical samples.

DNA samples were selected based on (i) previous results of laboratory routine diagnostic tests, epidemiological and clinical background, and (ii) availability of enough volume.

The clinical and epidemiological data considered related to leishmaniasis were fever, hepatosplenomegaly, skin lesion, living in or trips to an area of endemicity, exposure to sandfly bites, and immunosuppressed condition. The Ln-PCR data had greater relevance for the inclusion of cases. Raw epidemiological and clinical data are included in Data Set S1. A summary of the main characteristics to define the sample groups is detailed in Table 6.

### Leishmania DNA.

DNA was obtained from L. infantum culture of MHOM/Fr/LEM75 using the SpeedTools DNA/tissue DNA extraction kit (Biotools B&M Labs, S.A., Madrid, Spain) following the tissue protocol. The DNA concentration was measured with a NanoDrop 1000 spectrophotometer (Thermo Fisher Scientific, USA). For the preparation of *Leishmania* DNA dilutions, we assumed 200 fg of genomic DNA as the equivalent of one parasite (16).

### Diagnostic tests.

**(i) *Leishmania* culture in Novy-MacNeal-Nicolle (NNN) medium.** For NNN tube preparation, 10 g of bacteriological agar (Pronadisa, Laboratorios Conda, Spain) and 3 g of NaCl were dissolved in 450 mL of distilled water. After an autoclave step at 121°C and 1.5 atmospheres for 20 min, agar was stored at 4°C until use. Meanwhile, following the recommendations of the ISCIII animal facility, rabbit blood was collected into a sterile flask with glass beads, defibrinated by moderate agitation, and stored at room temperature (RT) until use on the same day or at 4°C for a maximum period of 18 h. Later, the bacteriological agar was melted in a microwave and cooled in a water bath at 56°C. Then, 100 mL of agar, 50 mL of defibrinated rabbit blood, and 10 mL of antibiotics (10,000 U/mL penicillin and 10,000 U/mL streptomycin) were mixed. Two milliliters of this mix was dispensed per tube and cooled on an inclined plane. Once the medium was gelled, the tubes were stored at 4°C until use. For parasite isolation by culture, 200 μL of the blood sample, bone marrow, or any other fluid was directly inoculated in an NNN tube at RT. Tissue biopsy specimens were previously ground using 300 μL of NET 10 as a diluent (0.1 M NaCl, 10 mM EDTA, and 10 mM Tris, pH 8) and a mechanical homogenizer, then 50 μL of this suspension was used to culture. The inoculated tubes were maintained at 27°C until weekly passage. In each passage, the total volume of inoculum was transferred to a new culture tube, analyzing 25 μL of the fluid by MO at 400×. The culture result was considered positive when promastigotes were observed. If after four passages (4 weeks) no parasites were observed, the culture result was considered negative.

**(ii) Ln-PCR.** DNA purification was performed using a SpeedTools DNA/tissue DNA extraction kit (Biotools B&M Labs, S.A., Madrid, Spain) following the manufacturer’s instructions, eluting DNA with water in a volume equal to the volume of the starting sample (up to 200 μL). Paraffin-embedded tissue samples were treated with 200 μL of 0.05% Tween 20 in phosphate-buffered saline, incubated at 65°C for 15 min, centrifuged for 5 min at 13,000 rpm, and cooled for 30 min at 4°C. Then, paraffin disks and aqueous supernatant were removed, and the sediment was processed as a fresh tissue sample. All tissue samples were treated with lysis solution and proteinase K at 56°C for 12 to 18 h in a shaking incubator. Purified DNA was analyzed immediately or stored at 4°C until use.

Ln-PCR was performed in two consecutive reactions following the protocol described by Cruz et al. (14), with minor modifications. The first reaction was performed in a final volume of 50 μL, using 10 μL of purified DNA with a final concentration of 1× reaction buffer (7.5 mM Tris-HCl [pH 9.0], 2 mM MgCl_2_, 5 mM KCl, and 2 mM [NH_4_]2SO_4_; Biotools B&M Labs, S.A., Madrid, Spain), 0.2 mM dNTPs each (Biotools B&M Labs, S.A., Madrid, Spain), 0.3 μM R221 primer (5′-GGTTCCTTTCCTGATTTACG-3′; Sigma, Spain), 0.3 μM R332 primer (5′-GGCCGGTAAAGGCCGAATAG-3′; Sigma, Spain), and 0.03 U/μL *Tth* polymerase (Biotools B&M Labs, S.A., Madrid, Spain), completing the final volume with DNA-free water. Amplification was performed in an AB 2720 thermocycler (Applied Biosystem, USA). The amplification conditions were denaturation at 94°C for 5 min, followed by 30 cycles of denaturation at 94°C for 30 s, annealing at 60°C for 30 s, and extension at 72°C for 30 s, with a final extension at 72°C for 5 min. The final holding temperature was 4°C. To perform the second reaction, 10 μL from a 1:40 dilution of the first amplification product was used (25 μL of the amplified product in 1 mL of water). This second reaction consisted of the final concentration of 1× reaction buffer (7.5 mM Tris-HCl [pH 9.0], 2 mM MgCl_2_, 5 mM KCl, and 2 mM [NH_4_]2SO_4_; Biotools B&M Labs, S.A., Madrid, Spain), 0.2 mM dNTPs each, 0.3 μM R223 primer (5′-TCCCATCGCAACCTCGGTT-3′; Sigma, Spain), 0.15 μM R333 primer (5′-AAAGCGGGCGCGGTGCTG-3′; Sigma, Spain), and 0.04 U/μL *Tth* polymerase (Biotools B&M Labs, S.A., Madrid, Spain), completing the 25-μL final volume with DNA-free water. The amplification conditions were similar to the first reaction except for the annealing step, which was increased to 65°C. The amplified products (25 μL) were visualized on a 1.5% agarose gel with 0.1× GelRed nucleic acid gel stain (Biotum, USA). In the first reaction, in samples with a high parasite load, an amplified product of 603 bp was observed. In the second reaction, in samples with both high and low parasite loads, the amplicon size was 358 bp.

**(iii) Serological diagnostic tests.** The rK39 ICT (Kalazar Detect rapid test, Inbios International, Inc., WA, USA) was performed according to the manufacturer’s instructions. This test was performed when blood samples were collected. Also, using the same sample, an in-house indirect immunofluorescence antibody test (IFAT) was performed when sufficient volume was available (31). The antigen was promastigotes from the L. infantum international reference strain MHOM/FR/78/LEM-75, samples and controls were diluted 2-fold from 1/40 to 1/160, and, to detect antibody binding, fluorescein isothiocyanate-conjugated goat anti-human immunoglobulin G (heavy and light chains; Jackson ImmunoResearch Laboratories, Inc., USA) was used. The threshold titer for positivity was 1/80.

### Index diagnostic tests.

**(i) LeishGelPCR.** Considering the composition of the Ln-PCRs, a third party (Biotools B&M Labs, S.A.) prepared individual ready-to-use tubes that consisted of dried reaction mixture and stabilizers in gel form of each reaction (www.biotools.eu). The DNA template and final volume of each reaction were the same as Ln-PCR. For the ready-to-use tube of the first reaction, 10 μL of DNA sample and 40 μL of PCR-grade water were added. To perform the second reaction, 10 μL of the amplified product of the first reaction was diluted in 1 mL of water and mixed by vortexing, and 25 μL of this solution was added to the ready-to-use tube of the second reaction. The amplification conditions were the same as the Ln-PCR except that five additional cycles of amplification were added in the first reaction. The amplified products were visualized as described above.

**(ii) Leish-qPCR.** We designed a duplex qPCR to simultaneously detect *Leishmania* DNA and mammalian DNA from conserved regions of the 18S rRNA gene. *Leishmania* detection was based on primers of the second reaction of Ln-PCR and the human DNA on PCR described in Norman et al. (32). Probes were designed using the OligoArchitect online design tool (Sigma, Merck) and visual examination of alignment of 18S rRNA sequences of different *Leishmania* species and main mammals (humans, mice, and dogs). Probe selection and validation were performed by *in silico* analysis using RealTimeDesign software (Biosearch Technologies). The Leish-qPCR was performed at a final volume of 30 μL that included 10 μL of purified DNA, 1× Quantimix Hotsplit probes kit (Biotools B&M Labs, S.A., Madrid, Spain), 0.7 μM and 0.33 μM R223 and R233 primers, respectively, 0.07 μM *Leishmania* probe (5′-CAL Fluor Red 610-AGACGAACTACAGCGAAGGCA-3′ BHQplus-2, Biosearch Technologies) for *Leishmania* detection, and 0.04 μM HUF (5′-GAGCCGCCTGGATACCGC-3′), 0.19 μM REV (5′-GACGGTATCTGATCGTCTTC-3′) primers, and 0.07 μM probe (5′-Quasar 670-TCGCTCTGGTCCGTCTTG-3′ BHQplus-2, Biosearch Technologies) for internal control (IC) detection. The cycling conditions were 5 min at 95°C, 3 cycles at 95°C for 10 s, 68°C for 15 s, 72°C for 20 s (with a touchdown in annealing temperature of one grade per cycle, up to 65°C), and 42 cycles at 95°C for 10 s, 65°C for 15 s, and 72°C for 20 s in a Rotor-Gene 6000 thermocycler (Corbett, Australia, and Qiagen, Germany). Leish-qPCR was positive when a threshold cycle (*C_T_*) value in the *Leishmania* channel was obtained. The reaction was negative only when a *C_T_* value in the IC channel was obtained; otherwise, the reaction was considered failed.

### Analytical sensitivity.

The analytical sensitivity or limit of detection (LOD) was determined by making 10-fold serial dilutions of *Leishmania* DNA in human DNA from whole blood of healthy volunteer donors. Assuming that 200 fg of *Leishmania* DNA was equivalent to 1 parasite (17), purified *Leishmania* DNA was diluted in human DNA solution at a concentration of 5 × 10^7^ parasite equivalents/μL and then down to 0.005 parasite equivalents/μL; these dilutions were used to determine the LOD of LeishGelPCR. To confirm the initial findings, repetitive testing from 0.5 to 0.005 parasite equivalents/μL was performed. Similarly, to determine the LOD of Leish-qPCR, purified *Leishmania* DNA was diluted at a concentration of 1 × 10^7^ to 0.0001 parasite equivalents/μL. To define the LOD value, repetitive testing from 0.1 to 0.0001 parasite equivalents/μL was performed. These values were multiplied by 10 (volume of DNA template) to convert to parasite equivalents/reaction.

### Clinical validation.

An exploratory performance evaluation was performed using 10 samples with positive results by Ln-PCR from different matrixes (bone marrow, whole blood, skin lesion biopsy specimens, and lymph node aspirates), five with positive results in both reactions of Ln-PCR and five with positive results in the second reaction only (Table S3). Specificity was determined with DNA from eight samples with negative results by Ln-PCR from samples from other parasitic infections (*Cryptosporidium* sp., Giardia lamblia, *Blastocystis hominis*, *Dientamoeba fragilis*, Plasmodium falciparum, Toxoplasma gondii, Trypanosoma cruzi, and *Babesia* sp.) and Mycobacterium tuberculosis (Table S4).

To determine diagnostic sensitivity and specificity, 200 samples from an at-risk population likely infected with *Leishmania* were tested by LeishGelPCR, 141 with a suspected VL and 59 with a suspected CL. Seven samples were excluded before running Leish-qPCR for volume availability issues (i.e., the sensitivity and specificity were assessed with 140 and 53 samples from VL and CL patients, respectively).

### Standard curve.

The standard curve was built with 10-fold serial dilutions of L. infantum DNA that were spiked on a pooled solution of human DNA from uninfected individuals. Eight dilutions were amplified in triplicate, starting from 1 × 10^4^ parasite equivalents/μL (i.e., 1 × 10^5^ parasite equivalents/reaction). Parasite equivalents/μL were converted to parasite equivalents/reaction by multiplying by 10 (volume of DNA template/reaction). The equation was calculated with *C_T_* values with a coefficient of variation lower than 25% after extrapolation to parasite equivalents.

### Data analysis.

Sensitivity (tested positive by index test/total of positive samples), specificity (tested negative by index test/total of negative samples), accuracy ([true tested positive + true tested negative by index test]/total samples), and 95% confidence interval (CI 95%) of index tests were estimated by binomial distribution (http://statpages.info/confint.html). Differences regarding the Ln-PCR as a standard were analyzed by McNemar’s test.

The LOD for LeishGelPCR was calculated as the highest dilution providing 95% positive results. For Leish-qPCR, the LOD was determined by PROBIT analysis. The receiver operating characteristic (ROC) curve and the corresponding area under the curve (AUC) were generated to confirm its accuracy and to determine the *C_T_* value under which its specificity could be 100%. The range and limit of quantification were determined through linear regression analysis. This process evaluated the slope of the regression line, the *y* intercept, and correlation coefficient, ensuring 99.9% efficiency. The extrapolation of *C_T_* values in parasite equivalents/reaction to estimate parasite load was performed by regression analysis using an equation derived of the standard curve. The median comparisons of *C_T_* values and the parasite load were performed by nonparametric tests. All these analyses were performed using IBM SPSS Statistics 25 software.

### Ethical clearance.

The use of samples was approved by the Research Ethics Committee of ISCIII, reference CEI PI 80_2016V3. As this study was retrospective, formal consent was not requested. The recommendations of the Ethics Committee that consented to the use of these samples for research were followed, ensuring the confidentiality of patients by anonymizing their data, in compliance with Spanish current legislation. All samples were residual specimens from the diagnostic routine and were kept in sample collections after anonymization.
