# Supplementary material for: Molecular Diagnosis of Leishmaniasis in Spain: Development and Validation of Ready-To-Use Gel-Form Nested and Real-Time PCRs To Detect Leishmania spp
Source: Microbiol Spectr. 2023 Apr 4;11(3):e03354-22. doi: 10.1128/spectrum.03354-22 (PMC10269443; doi:10.1128/spectrum.03354-22)
Supplement: Supplemental file 2 — Fig. S1 to S3 and Tables S1 to S4. Download spectrum.03354-22-s0001.pdf, PDF file, 1.9 MB [file spectrum.03354-22-s0001.pdf]

SUPPLEMENTAL MATERIAL

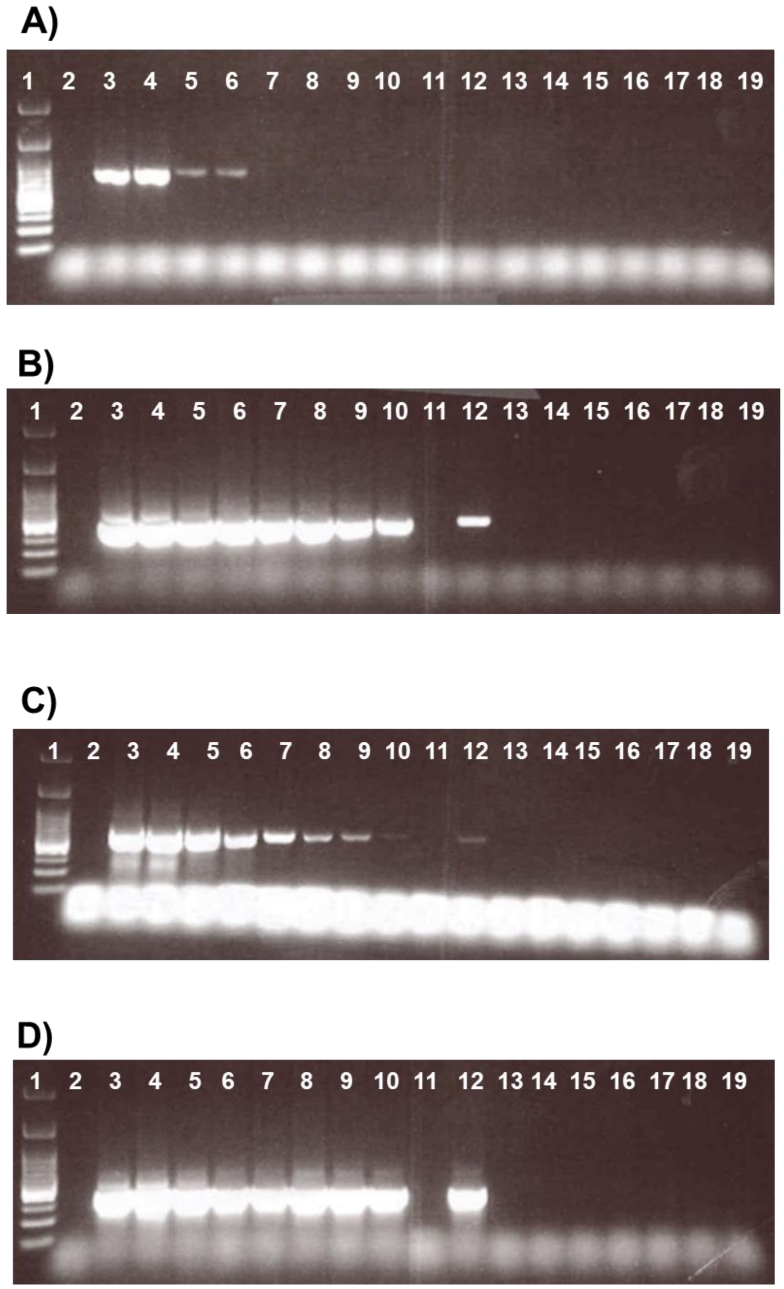

Figure S1. Analytical sensitivity of first and second PCR reaction of Ln-PCR (A & B) and LeishGelPCR (C & D) using spiked samples from $5x10^6$ to 0.05 parasites/reaction. Line 1, 100 bp ladder; line 2, not template control; line 3, $5x10^6$; line 4, $5x10^5$; line 5, $5x10^4$; line 6, $5x10^3$; line 7, 500; line 8, 50; line 9, 5; line 10, 0.5; line 11, 0.05 parasites/reaction; line 12, positive control, the size of amplified product is 600 bp in the first reaction, and 330 bp in the second reaction.

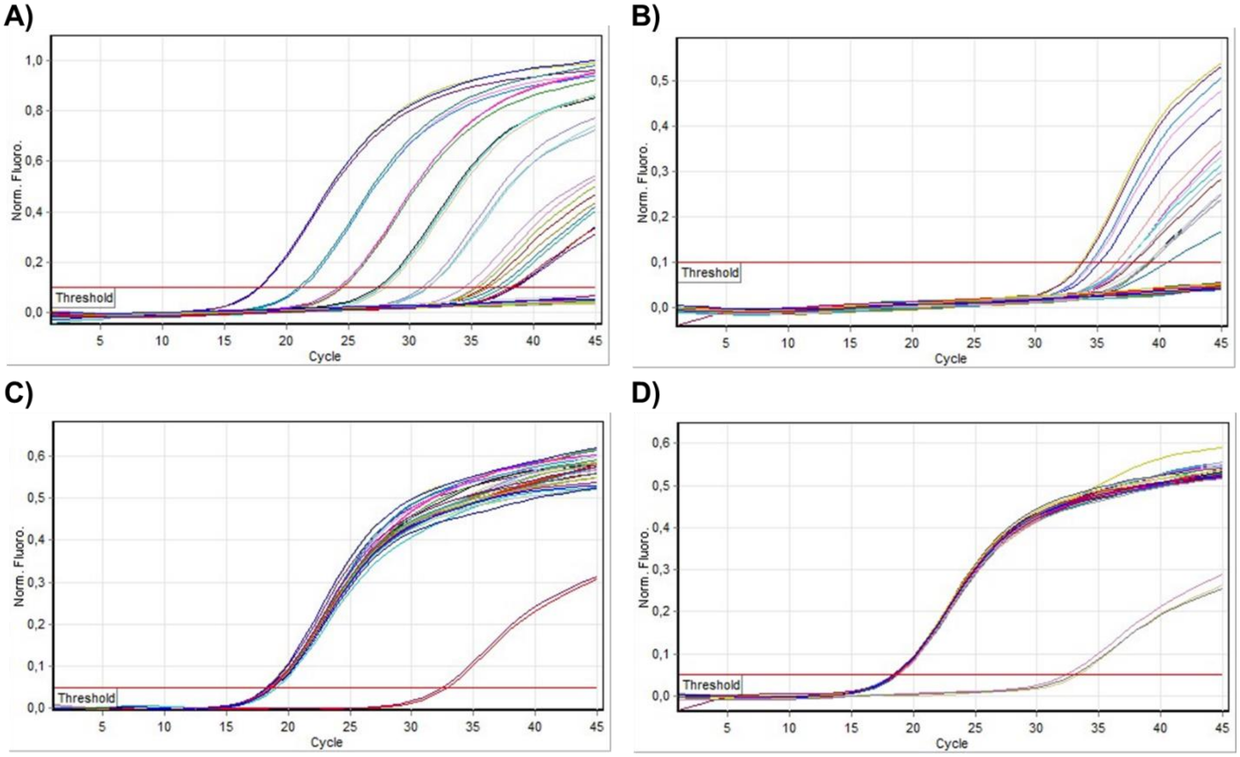

Figure S2. Analytical sensitivity of Leish-qPCR using spiked samples: A) Blood samples with DNA *Leishmania* adjusted at $1x10^6$ to 0.001 parasites equivalents/reaction tested in triplicate. B) Blood samples with *Leishmania* DNA adjusted at 1 to 0.01 parasites equivalents/reaction tested in quintuple. C & D) Detection of the internal control.

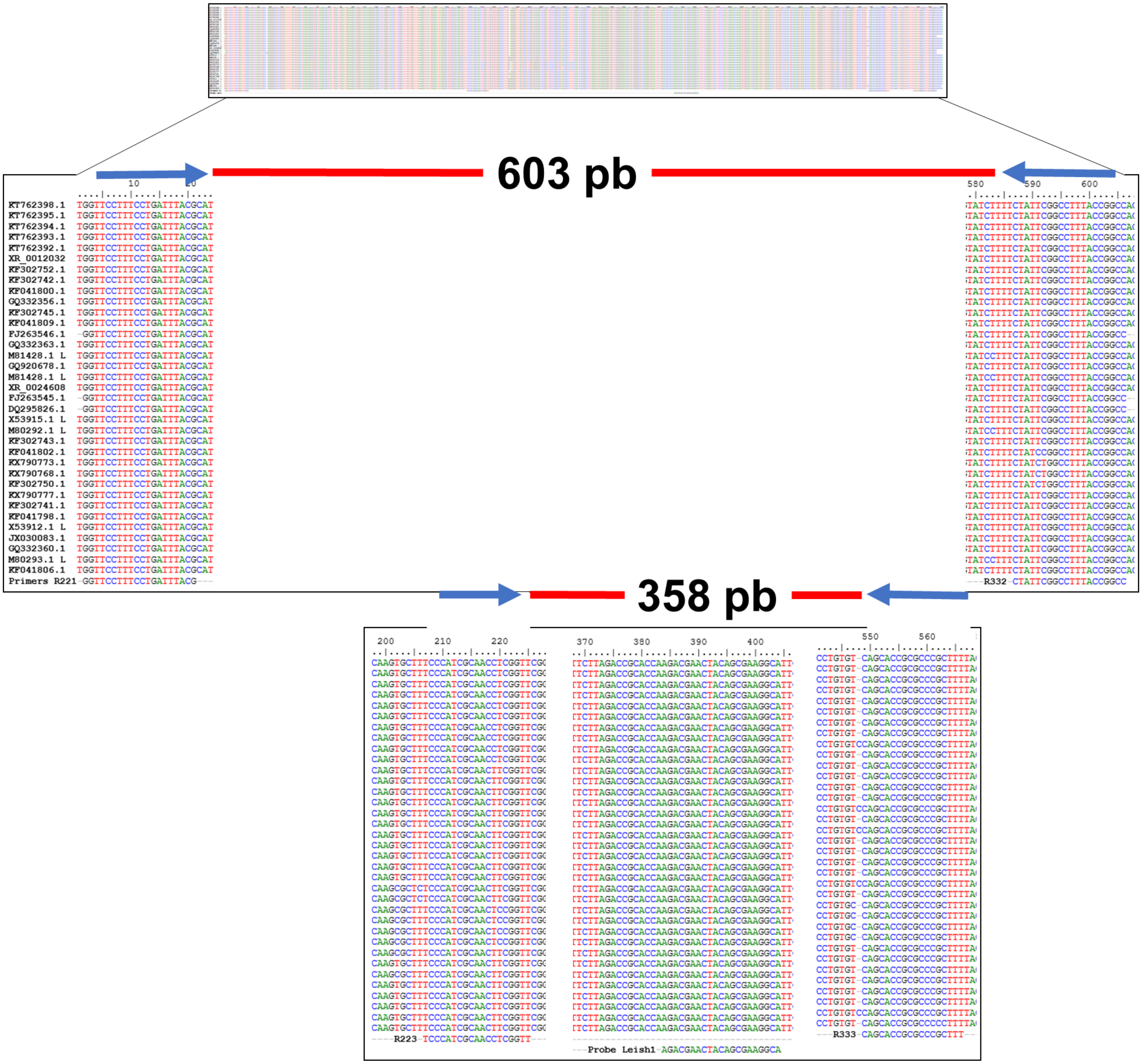

Figure S3. Alignment of different *Leishmania* species. Sequences were downloaded from Gene bank. Sequences of R333 and R332 are in reverse complementary. Superior part is the product can be obtained in the first reaction of Ln-PCR and LeisGelPCR. Lower part is the product of the second reaction of nested PCRs. Also, it is the product of the Leish-qPCR. *L. chagasi*: KT762398.1; KT762395.1; KT762394.1; KT762393.1; KT762392.1. *L. infantum*: XR_001203206.1; KF302752.1. *L. donovani*: KF302742.1; KF041800.1; GQ332356.1. L. tropica: KF302745.1; KF041809.1; FJ263546.1; GQ332363.1. *L. aethiopia*: M81428.1; GQ920678.1; M81428.1. *L. major*: XR_002460808.1; FJ263545.1; DQ295826.1; X53915.1. *L. brasiliensis*: M80292.1. *L. guyanensis*: KF302743.1. *L. equatorensis*: KF041802.1; KX790773.1. *L. colombiensis*: KX790768.1; KF302750.1. *L. enriettii*: KX790777.1; KF041798.1. L. mexicana: KF302741.1. *L. amazonensis*: X53912.1; JX030083.1. *L. mexicana*: GQ332360.1; M80293.1; KF041806.1.

Table S1. Comparison of the limit of detection of Ln-PCR and LeishGelPCR

**N parasites per reaction**	**Ln-PCR**		**LeishGelPCR**	
	**1st reaction**	**2nd reaction**	**1st reaction**	**2nd reaction**
	**N Pos reactions per total repetitions (%)**	**N Pos reactions per total repetitions (%)**	**N Pos reactions per total repetitions (%)**	**N Pos reactions per total repetitions (%)**
**5**	0/10 (0)	10/10 (100)	8/10 (80)	10/10 (100)
**0.5**	0/10 (0)	8/10 (80)	4/10 (40)	10/10 (100)
**0.05**	0/10 (0)	4/10 (40)	0/10 (0)	1/10 (10)

Pos, positive.

Table S2. Limit of detection (LOD) of Leish-qPCR

**Parasites per reaction**	**Average of C_T**	**Positive reactions per total repetitions (%)**	**Expected positive reactions**	**Likelihood (%)**
**1**	34.66	8/8 (100)	8	100
**0.1**	37.23	8/8 (100)	7	91.6
**0.01**	37.90	5/8 (62.5)	6	76.5
**0.001**	39.26	2/8 (25)	2	20.9

At 95% of probit likelihood, the LOD of Leish-qPCR in terms of Ct value was 37.084 (CI 95%: 34.33 – 37.74), so in terms of parasites/reaction, the LOD was 0.2 (CI 95%: 0.1 – 1.1).

Table S3. Comparison of Ln-PCR, LeishGelPCR and Leish-qPCR using clinical samples from leishmaniasis patients.

**Sample**		**Ln-PCR**				**LeishGelPCR**				**Leish-qPCR**
**Code**	**Matrix**	**1st reaction**		**2nd reaction**		**1st reaction**		**2nd reaction**		
		**10 µL**	**20 µL**	**10 µL[a]**	**20 µL[a]**	**10 µL**	**20 µL**	**10 µL[a]**	**20 µL[a]**	**10 µL**
S01	FTB	**P**	**P**	**P**	**P**	**P**	**P**	**P**	**P**	**P**
S02	LNA	**P**	**P**	**P**	**P**	**P**	**P**	**P**	**P**	**P**
S03	BM	**P**	**P**	**P**	**P**	**P**	**P**	**P**	**P**	**P**
S04	WB	**P**	**P**	**P**	**P**	**P**	**P**	**P**	**P**	**P**
S05	BM	**P**	**P**	**P**	**P**	**P**	**P**	**P**	**P**	**P**
S06	BM	N	N	**P**	**P**	**P**	**P**	**P**	**P**	**P**
S07	FTB	N	N	**P**	**P**	**P**	**P**	**P**	**P**	**P**
S08	WB	N	N	**P**	**P**	**P**	**P**	**P**	**P**	**P**
S09	FTB	N	N	**P**	**P**	N	N	N	**P**	**P**
S10	FTB	N	N	N	**P**	N	N	**P**	**P**	**P**

BM, bone marrow; FTB, fresh tissue biopsies; LNA, lymph node aspirate; WB, whole blood; P, positive; N, negative.
[a]The 2nd reaction was carried out with 10 µL of the 1:40 dilution of the amplified product of the 1st reaction.

Table S4. Comparison of Ln-PCR, LeishGelPCR and Leish-qPCR using DNA samples from uninfected individuals and other pathogens

Clinical status	Matrix of sample	N samples	N positive reactions by Ln-PCR	N positive reactions by LeishGel PCR	N positive reactions by Leish-qPCR
Uninfected					
CL suspicion	FTB	2	0	0	0
VL suspicion	BM	4	0	0	0
	WB	1	0	0	0
	LNA	1	0	0	0
Other parasite infections					
Cryptosporidium sp	Stool	2	0	0	0
Giardia lamblia	Stool	2	0	0	0
Blastocystis hominis	Stool	2	0	0	0
Dientamoeba fragilis	Stool	2	0	0	0
Plasmodium falciparum	WB	2	0	0	0
Toxoplasma gondii	CSF	1	0	0	0
Trypanosoma cruzi	WB	2	0	0	0
Mycobacterium tuberculosis	Culture	2	0	0	0

BM, bone marrow; CSF, cerebrospinal fluid; FTB, fresh tissue biopsies; LNA, lymph node aspirate; WB, whole blood.
